# China’s 2026 revision of National Ambient Air Quality Standards: A critical step toward health-based air pollution governance

**DOI:** 10.1016/j.xinn.2026.101463

**Published:** 2026-06-03

**Authors:** Jing Cai, Haidong Kan

**Affiliations:** 1 School of Public Health, Key Lab of Public Health Safety of the Ministry of Education and NHC Key Lab of Health Technology Assessment, Fudan University, Shanghai 200032, China

**Figure:**
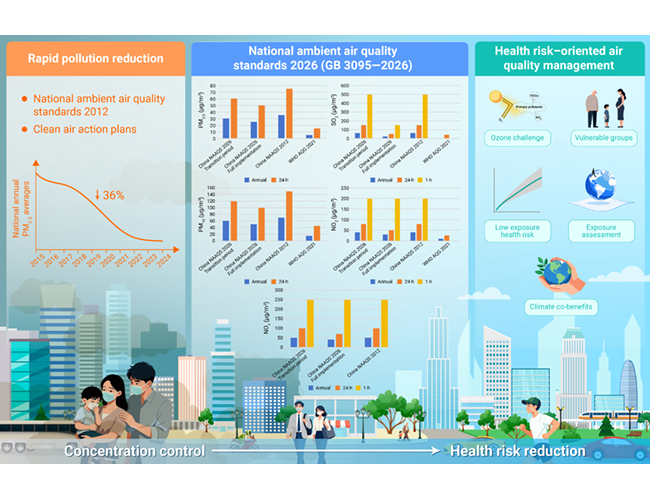

## Tightening standards in a changing regulatory context

Over the past decade, China has experienced one of the most rapid large-scale improvements in air quality ever documented. Between 2015 and 2024, national average fine particulate matter (PM_2.5_) concentrations declined by approximately 36%.^1^ This achievement has been widely regarded as a major large-scale air pollution mitigation effort globally. Since the implementation of the first national Clean Air Action Plan in 2013, regulatory priorities and performance evaluations for local governments have emphasized lowering annual average concentrations. As pollution levels decline, the challenge policymakers face has fundamentally evolved: the marginal health benefits of further improvement become increasingly dependent on the management of health risks at lower concentrations.

Against this backdrop, China released the revised National Ambient Air Quality Standards (NAAQS; GB 3095-2026) in February 2026,^2^ representing the most significant update since the 2012 revision that first incorporated PM_2.5_. The new standard significantly tightens concentration limits for major pollutants (Table 1), including PM_2.5_, inhalable particulate matter (PM_10_), sulfur dioxide, nitrogen dioxide, and nitrogen oxide. For instance, the annual PM_2.5_ limit is lowered from 35 to 25 μg/m^3^ for class II areas (representing general residential areas), accompanied by stricter daily limits (lowered from 75 to 50 μg/m^3^). In addition, the revision adopts a phased implementation strategy, introducing transitional targets (e.g., interim class II limits of 30 [annual] and 60 [daily] μg/m^3^ for PM_2.5_) between 2026 and 2030 before full nationwide enforcement beginning in 2031. This 5-year transitional period (2026–2030) aligns closely with China’s 15th Five-Year Plan, reflecting strong coordination between air quality standard implementation and national socioeconomic planning cycles.

**Table 1:** Comparison of current NAAQS (GB3095-2026, Class II areas) with previous NAAQS (GB3095-2012, Class II areas), WHO AQG 2021, and standards of selected countries

Parameters		China NAAQS (GB3095-2026)^a^		China NAAQS (GB3095-2012)	World Health Organization AQG 2021					US	EU	India^b^
		Transition period (2026–2030)	Full implementation (2031 onwards)		AQG	IT-4	IT-3	IT-2	IT-1			
PM_2.5_ (μg/m^3^)	Annual	30	25	35	5	10	15	25	35	9	10	40
	24-h	60	50	75	15	25	37.5	50	75	35	25	60
PM_10_ (μg/m^3^)	Annual	60	50	70	15	20	30	50	70	–	20	60
	24-h	120	100	150	45	50	75	100	150	150	45	10
SO_2_ (μg/m^3^)	Annual	60	20	60	–	–	–	–	–	–	20	50
	24-h	150	50	150	40	–	–	50	125	–	50	80
	1-h	500	150	500	–	–	–	–	–	197	350	–
NO_2_ (μg/m^3^)	Annual	40	30	40	10	–	20	30	40	100	20	40
	24-h	80	50	80	25	–	–	50	120	–	50	80
	1-h	200	200	200	–	–	–	–	–	188	200	–
NO_X_ (μg/m^3^)	Annual	50	40	50	–	–	–	–	–	–	–	–
	24-h	100	70	100	–	–	–	–	–	–	–	–
	1-h	250	250	250	–	–	–	–	–	–	–	–
O_3_ (μg/m^3^)	Peak season	–	–	–	60	–	–	70	100	–	–	–
	MDA8	160	160	160	100	–	–	120	160	137	120	100
	1-h	200	200	200	–	–	–	–	–	–	–	180

NAAQS, National Ambient Air Quality Standards; AQGs, air quality guidelines; IT, interim target; US, United States; EU, European Union; PM_2.5_, fine particulate matter; PM_10_, inhalable particulate matter; SO_2_, sulfur dioxide; NO_2_, nitrogen dioxide; NO_X_, nitrogen oxides; O_3_, ozone; MDA8, maximum daily 8-h average of ozone.

Carbon monoxide is not presented in the table because the China NAAQS (GB 3095–2026) did not revise the corresponding standard.

a The China NAAQS defines two classes of standards: class I applies to special regions (including national parks), while class II applies to all other areas.

b The standards presented for India apply to industrial, residential, rural, and other areas.

## From rapid pollution reduction to health-risk-oriented management

First formulated in 1982, China's NAAQS built the foundational framework for national air quality management. Subsequently, the 1996 revision improved indicator systems and regulatory normalization. The 2012 revision marked a turning point by incorporating PM2.5, underpinning three consecutive clean air action plans that drove substantial air quality improvements.

However, accumulating evidence indicates that adverse health effects persist even at low PM_2.5_ levels, with no clear threshold.^3^ As many cities approach previous compliance targets, the 2012 standards are losing their guiding function for further health protection. By 2024, the national annual mean PM_2.5_ concentration had fallen to 28 μg/m^3^,^1^ already below the transitional annual target of 30 μg/m^3^ for the 2026–2030 period. This progress reflects the cumulative effects of sustained air pollution control efforts; however, attainment at the national average level does not obviate the need for interim regulatory thresholds, given persistent and substantial regional heterogeneity in exposure levels. The transitional target therefore may function as a coordination mechanism that drives faster improvements in higher-exposure regions, thereby promoting regional air quality convergence. Overall, the 2026 revision signals a shift in environmental governance, in which regulatory effectiveness is increasingly judged not only by meeting concentration limits but by reducing health risks.

## Aligning health protection ambition with regulatory feasibility

In 2021, the World Health Organization (WHO) updated its global air quality guidelines (AQGs), lowering the recommended annual PM_2.5_ concentrations to 5 μg/m^3^ in response to strengthened causal evidence linking long-term exposure to multiple health outcomes.^4^ Regulatory agencies in the United States and European Union have also moved toward stricter standards, although the process—particularly in the United States—has reflected evolving scientific assessments within a complex regulatory and political context.

As shown in Table 1, NAAQS differs across countries, reflecting path-dependent regulatory development shaped by governance capacity, technological readiness, and accumulated pollution burdens. China’s revised NAAQS sets an annual mean PM_2.5_ limit of 25 μg/m^3^ for class II areas—tighter than India’s current standard (40 μg/m^3^) for industrial, residential, rural, and other areas yet higher than those in the United States (9 μg/m^3^) and the European Union (10 μg/m^3^). Notably, the revised Chinese standard aligns with WHO interim target-2, designed to support countries transitioning from relatively high pollution levels toward progressively lower-risk exposure regimes.

China’s revised PM_2.5_ standard thus occupies an intermediate position within the global spectrum, balancing health ambition with implementation feasibility. Given its population scale, industrial structure, and regional heterogeneity in pollution profiles, abrupt adoption of ultra-stringent limits may pose substantial implementation challenges. The phased tightening strategy thus reflects an adaptive governance approach integrating scientific evidence with developmental realities. Considering that China and India together account for nearly one-third of the global population, incremental yet enforceable improvements in such large economies may yield greater reductions in global exposure than aspirational but impractical targets. In this sense, China’s pathway may offer a potentially transferable model for populous countries transitioning toward health-oriented air quality management.

## Persistent challenges: Ozone health risk management

While particulate matter pollution has declined nationwide, ozone has emerged as an increasingly prominent challenge in China. Unlike particulate matter, ozone formation arises from nonlinear photochemical interactions among precursor emissions and meteorological conditions, necessitating coordinated management of multiple pollutants.

The revised Chinese standards retain existing ozone limits based on peak exposure metrics, including a maximum daily 8-h average and a 1-h limit (Table 1). Similar to regulatory approaches in the United States, Europe, and India, legally binding long-term ozone exposure limits remain absent. This reflects persistent scientific and regulatory challenges: ozone concentrations are strongly influenced by meteorology, hemispheric transport, and climate-driven processes beyond direct domestic control. Importantly, China has already implemented a series of coordinated precursor control strategies targeting both nitrogen oxides and volatile organic compounds, including tightened industrial emission standards and fuel quality improvements. These measures, together with synergistic PM_2.5_-ozone co-control strategies, have partially mitigated ozone increases in certain regions.

As evidence linking middle- or long-term ozone exposure to chronic health outcomes continues to grow, developing enforceable frameworks capable of addressing cumulative ozone risk represents one of the central unresolved challenges in global air quality governance. In this context, future revisions of China’s air quality standards may consider incorporating peak-season or other long-term metrics to better capture cumulative exposure risks.

## Implications for environmental health science

Beyond regulatory adjustment, China’s new standards carry profound implications for environmental health research. Continued tightening will shift population exposure into lower concentration ranges, creating opportunities to characterize exposure-response relationships under low-exposure conditions—an area where existing Chinese evidence remains limited. Future studies are needed to assess potential nonlinearities and refine risk estimates relevant to subsequent standard revision. Lower exposure levels may also impose stricter requirements on exposure assessment. Under low-exposure conditions, even minor measurement errors may substantially bias effect estimates, underscoring the need for advanced approaches, such as dense low-cost sensor networks and machine-learning-based spatiotemporal models, to improve exposure characterization and detect subtle health effects. Meanwhile, research priorities should expand beyond mortality outcomes toward broader measures of disease burden, including chronic diseases, neurodevelopmental effects, reproductive health, and aging-related vulnerability. As China’s population ages, identifying susceptible subgroups and addressing environmental health inequities may become increasingly central to policy translation.

Overall, as air quality improves, research priorities of environmental health are shifting from documenting pollution-related harm toward quantifying residual risks at low exposure, refining exposure metrics, and incorporating heterogeneity and uncertainty into policy-relevant assessments. In this context, health-risk-oriented management is emerging as a central organizing principle of next-generation air quality governance, placing methodological innovation at the interface of epidemiology, exposure science, and regulatory decision-making.

## Linking clean air and climate goals

Besides air pollution, climate change represents a major public health challenge. China has committed to peaking carbon emissions before 2030 and achieving carbon neutrality by 2060. Given their shared sources, continued air quality improvement will inherently support low-carbon transitions. Meanwhile, previous studies suggest that carbon neutrality could reduce national PM_2.5_ levels toward the 2021 WHO interim target-4 guideline (10 μg/m^3^), generating substantial health co-benefits.^5^ Integrating air quality management with climate policy therefore offers a critical pathway to maximize health gains.

Overall, the 2026 revision marks both an achievement and a new starting point toward the next generation of air quality management. As countries confront the dual challenges of environmental sustainability and public health protection, China’s transition illustrates a health-driven yet feasibility-constrained regulatory paradigm, in which continuous tightening is guided by epidemiological evidence while calibrated to economic and institutional capacity. Rather than pursuing immediate attainment of ultra-stringent targets, this approach emphasizes cumulative health gains through enforceable and adaptive pathways, offering a transferable model for other large and rapidly developing economies. Achieving these goals will depend on continued high-quality environmental health research—particularly at low-exposure levels—and coordinated implementation alongside China’s dual-carbon objectives.

## Funding and acknowledgments

This work was supported by a grant from the 10.13039/501100002855Ministry of Science and Technology of the People's Republic of China (2022YFC3703002).

## Declaration of interests

The authors declare no competing interests.
